# Supplementary material for: Patterns of Intron Gain and Loss in Fungi
Source: PLoS Biol. 2004 Nov 30;2(12):e422. doi: 10.1371/journal.pbio.0020422 (PMC532390; doi:10.1371/journal.pbio.0020422)
Supplement: Table S1 — Also available at http://genes.mit.edu/NielsenEtAl/. (4.3 MB ZIP). [file pbio.0020422.st001.zip › NielsenEtAl/html/44.html]

AN6897.1.NCU00436.1.MG05209.1.FG04113.1


```
 CLUSTAL W (1.82) Multiple Sequence Alignments - Introns Inserted


Sequence 1: NCU00436.1	918 aa
Sequence 2: FG04113.1	898 aa
Sequence 3: MG05209.1	911 aa
Sequence 4: AN6897.1	935 aa
Alignment Length: 950 aa
Number Identitical Residues: 406 aa
Alignment Score (without introns) 19930


MG05209.1 	MSQDSAHSSDATGPAPGARSNGE----GSSTATNETPSSVPSGPPSYMTVGTGSTSEHAH
NCU00436.1	MSQD-YYPS--KGKAPQQHEDGEPREFDDGAEHHPQVPGAPTT-PAYMTVGTGSTSQHAA
FG04113.1 	MSQD-YFSGNGKGKARMQQETDSDGDHANAPNSNQTSPPADSSRVQYMTVGNGMASEHAA
AN6897.1  	MSQE-YFPG--KGGSSAGEGSSR----NPLDEKPEESPFAPRLPPSYMTVGNGSTSESAT
          	***:  ...  .* :   .  .               . .      *****.* :*: * 

MG05209.1 	RLQALLDNDSGYGGSIAGDGAPLIGASGDHHQALVDPDRRMQ----------------AG
NCU00436.1	RLQAMLDNDSGYGGSIAGDSR-----APSHWDSAVHHDSPLPTPTTATHDDDANRRLQAG
FG04113.1 	RLQNMLDIDSGYGGSIAGDEQRSM-ASAAAWDMSMHHDRPAS-----------------G
AN6897.1  	ALMNSLHADSGYGGSVADGESGASWRSDLMVDRPTPAHTPTHPG-QWNPAAEHEKQIVAS
          	 *   *. *******:*..       :    :     .    .    .   . .    :.

MG05209.1 	AIHQLYY~NSHRVALARSINLAIQLLKGLREMNAKWPAHYPSVQG--TDTPTSPRPGNLR
NCU00436.1	AVHQLWY~NQHRVTLGRSINTVVELLKKLQEMNVTWPAHYPSVQRAVLDEPNNYGPPGLH
FG04113.1 	AVHQMWY~NAHRATLGRSINKVLELLDNLREMNSSWPAHYPSVQRTRRD-SSEAPRPGLA
AN6897.1  	HVHQLLY2NSNRTKLARAISRTIETLKELQDMNRQWPAHYPSVQN-LPDSPSDR--PSLQ
          	 :**: * * :*. *.*:*. .:: *. *::**  *********    * ...    .* 

MG05209.1 	HSFSSVGEFAASATAASHPRELRRSLTSVEDIGVQDAESSKAAERRQAAAEPRLVSPQIS
NCU00436.1	RSSTMGADFPPPP----SPHSLRRSMTTGDDHAE--PESSRAAERRNTSSEPRLVSPQIA
FG04113.1 	HAYTGMDGIPSSP----HHPALRRSMTTVEDNS---AESSRAAENRSTP-EPRLVSPQIA
AN6897.1  	QSQSFFGDAAAHNDGLARPGSLRRAATMPTNEEL--AESSATAERRAPP-EPRLMSPQIA
          	:: :     ..   . :    ***: *   :     .*** :**.* .. ****:****:

MG05209.1 	REFSILKLDLKLGSLHQTELVHSLEKGSVASLLDGKIGSSIKHLQSLRERIEDTSSKVLV
NCU00436.1	QEFSVLKLDLNLGSLHQADLVHSLQKESVASLLDGKIRSSIKHLYSLRERIEDTSSKVLV
FG04113.1 	QEFSVLKLDLKLGALHQTELVHSLEKSSIAALLDGKISTSIKHLIALRERIEDTASKVLI
AN6897.1  	QEFSILKLDLKLGALSQAELVHSLEKSSIASLLDGKIGQSIKHLLSLRDRIEDTSSKVLI
          	:***:*****:**:* *::*****:* *:*:******  ***** :**:*****:****:

MG05209.1 	TGDLNAGKSAFCNALLRRKILPEDQQPCTSIFCEVLDARENSGLEEVHAVHKEAIYDRND
NCU00436.1	TGDLNAGKSTFCNALLRRKVLPEDQQPCTSIFCEVLDARENGGIEEVHAVHRDAIYDRHD
FG04113.1 	TGDLNAGKSTFCNALLRRKVLPEDQQPCTAIFCEVLDARENSGVEEVHAVHKDVPYDRND
AN6897.1  	TGDLNAGKSTFCNALLRRKVLPEDQQPCTSIFCEVLDARENGGLEEVHAVHKDRQYNRND
          	*********:*********:*********:***********.*:*******::  *:*:*

MG05209.1 	ESTYDVFSLSDLEKIVIDNETYLQCKVYVKDVRTIDESLLNNGVVDIALIDAPGLNSEMT
NCU00436.1	EATYDVYSLKELERIVTDNETYQQCKIYIRDARTIDESLLNNGVVDIALIDAPGLNMDTT
FG04113.1 	ESTYDVFSLKELENIVIDNETYTQCKIYVKDSRSIDESLLNNGVVDIALIDAPGLNSDTT
AN6897.1  	ETTYDVYALSELENIVIDNTKYMQCKVYVKDVRTIDESLLNNGVVDIALIDAPGLNSDSL
          	*:****::*.:**.** ** .* ***:*::* *:********************** :  

MG05209.1 	KTTAVFARQEEIDVVVFVVSAANHFTISAQDFISVAAAEKAYLFIVVNQFDNIRDKERCQ
NCU00436.1	KTTAIFARQEEIDVVVFVVSATNHFTQTATEFIRAAAAEKAYLFIVVNGFDTIRDKERCQ
FG04113.1 	KTTAIFARQEEIDVVVFVVSAANHFTQSAKEFIWAAAAEKAYIFIVVNGFDVIRDKKRCE
AN6897.1  	KTTAVFARQEEIDVVVFVVSAANHFTLSAQDFIWNAAHEKAYMFMVVNGFDQIRDKQRCQ
          	****:****************:**** :* :**  ** ****:*:*** ** ****:**:

MG05209.1 	KMILDRVKMMSPRTYKEASELVHFVSSNAVPVAPPPPPPGGSGGSGSSSGGSGGD-----
NCU00436.1	KLILNQVRGLSPATHKEADELVHFVSSTAIPMAPSPPGGSYGGGSGSASGGGGGD-----
FG04113.1 	KMVLDQVAGLSPRTHKENSELVHFVSSNAIPVGPSPPGGPSGGGS---SGGGSGD-----
AN6897.1  	RMILDQIKKLSPRTYKEAAELVHFVSSNAVPVAPQIPVAGPGGGGGGGGGGSDPNGNDWP
          	:::*:::  :** *:**  ********.*:*:.*  *    .**.....**.. :... .

MG05209.1 	--GDDDPNEEDSKGKGKGKDKEKIRDFENLEQSLRRFVLEKRSRSKLAPAKNYLLNILAD
NCU00436.1	--DGDDDDP-----KGKGKKKEMARDFSNLEQSLRRFVLEKRARSKLAPAKTYLTNILND
FG04113.1 	--PGDGDD------KGKGKDLDKVRDFEALEQSLRRFVLEKRARSKLAPARTYLLNILND
AN6897.1  	SGDDDEDKPEDKKGKGKEKEKEKIQDFENLESSLRRFVLEKRSRSKLAPARTYLLNLLAD
          	:. .*  . .....*** *. :  :**. **.**********:*******:.** *:* *

MG05209.1 	INALAQVNVEVAQSELDRVNQELAEIEPMLESGRKASSQVDEQMDKSIEKTCSEVYEHTR
NCU00436.1	VHVLATVNQEVAQSEFDRMNQELQKLEPELERSKQAKQEINEKVDQTIEETCQEIYEFSR
FG04113.1 	VNTLATVNQEVAQSELDRVTQELKELEPQLESSRRARSEVSEKVDANVEDTCKEIYDYTR
AN6897.1  	INALAAVNRDVADKELQNVMKELADLEPAYENGKKKKIQLQEEVDKCIDDSCEDVYKHTR
          	::.** ** :**:.*::.: :** .:**  * .::   ::.*::*  ::.:*.::*..:*

MG05209.1 	STLSFTIQNIGDADLGVRYPGIWGSFDYAEQLIEAMLAQISDAVAACEDHARARTVSGVN
NCU00436.1	NTISSSIEHAGDGNFGIPYPGLFSAFEYAEELKEAMLSQIAGSVTQCEEHARARTVDGVN
FG04113.1 	NTLNSAILHSGSSHYDVPYPGIWSAFQYAEDLKEAMLSRIADSVANCEEYGRGKTVYGVN
AN6897.1  	TTLTSAIANVSEANLGVEYPGIFSAFQYAEDLKFAMLEHISASVAGCEDYARTKTAQGVG
          	.*:. :* : .... .: ***::.:*:***:*  *** :*: :*: **::.* :*. **.

MG05209.1 	TIKQLGLLHLG-DEYADLSFRPDVMFRR-KRDVLARQADIPTEIWDFIDWNTLIRRQEKV
NCU00436.1	MIKQLGIMHLG-NEYTDLAFRPDVMFRR-KKDALARQVDIPTELWDFVDWSTVLQKEEK-
FG04113.1 	AIKQLGILHVG-DEFQNLQFRPDVMFRR-KRDALARQVDIPTEMTDFVDWTTLLQRQEKF
AN6897.1  	FIQNLGLLHIGEDKFTAPTFRADLMFRRGRRHALARQVDTEVELLDFFDITGLWERQEKL
          	 *::**::*:*.:::    **.*:****.::..****.*  .*: **.* . : .::** 

MG05209.1 	AGTGMALTVVTAVGGQLVVGYGWMDHALRAARVLGNDNLRRLIIPGILVAA1VAASAFVL
NCU00436.1	--AGMALTVAGVVGTSVLSGYSQMNLALRAAQILGTDNVRRLIIPGLIAAA1VAATYYVL
FG04113.1 	AGTGMALTVAGAVVPRMIGMNTWMDQAITATKILSNENLRQLIIPGILVAA1AAASAYVL
AN6897.1  	AGTGMAMTAVTVLGGRAFGGISWLDSAFSAAKFLGPNHMRRLLLPGIVAAA1AITVAYVL
          	:.:***:*.. .:    .     :: *: *::.*. :::*:*::**::.** . :  :**

MG05209.1 	NSIPQSLPHRLSAKVSSHLAATDYVHLNSARISGSVRKVLRIPADSVRVGLRRSVEQLGT
NCU00436.1	NQIPHSLPHRLKEKISTQLAAIDYVHSNSIRISGSVRKVLRFPADSLRVGLQRSVEKLDG
FG04113.1 	QQIPNSLPPRLATKISTQLAEMDYVHANAARISSSVRKVLRIPADNLRVGLDQSLKDLSA
AN6897.1  	SSIPQTLPPRLSRKIAATLSEMDYVHSNSTRISSEVRRILRLPANNLQNSLAQDVEDLGR
          	..**::** **  *::: *:  **** *: ***..**::**:**:.:: .* :.::.*. 

MG05209.1 	RRDETLRISAESAVALKYFGNLVRDSAKHRATIDEVDLEAHGAA--------
NCU00436.1	RRNETLRVKAESDVALKYFANLVRESAHQRRVVEAVDLDGHPPGLAGIGGFH
FG04113.1 	KREETVKVKGESERASRYFLNLVRQSDDQRVKVEAVDLETPPPGMH------
AN6897.1  	RKQEVSKTKQESEVASKYFANLFRESTDNRTSVENIDLDGPLPGGLGAYEA-
          	:::*. : . **  * :** **.*:* .:*  :: :**:   ..  .
```
